# Is expert opinion reliable when estimating transition probabilities? The case of HCV-related cirrhosis in Egypt

**DOI:** 10.1186/1471-2288-14-39

**Published:** 2014-03-17

**Authors:** Anthony Cousien, Dorothée Obach, Sylvie Deuffic-Burban, Aya Mostafa, Gamal Esmat, Valérie Canva, Mohamed El Kassas, Mohammad El-Sayed, Wagida A Anwar, Arnaud Fontanet, Mostafa K Mohamed, Yazdan Yazdanpanah

**Affiliations:** 1 IAME, UMR 1137, INSERM, F-75018 Paris, France; Univ Paris Diderot, Sorbonne Paris Cité, F-75018 Paris, France; 2 EA2694, Université Lille Nord de France, Lille, France; 3 Inserm U995, Université Lille 2 – Lille Nord de France, Lille, France; 4 Department of Community, Environmental and Occupational Medicine, Faculty of medicine, Ain Shams University, Cairo, Egypt; 5 Department of Endemic Medicine and Hepatology, Faculty of Medicine, Cairo University, Cairo, Egypt; 6 Service des Maladies de l'Appareil Digestif et de la Nutrition, Hôpital Huriez, CHRU Lille, France; 7 National Hepatology and Tropical Medicine Research Institute, Cairo, Egypt; 8 Conservatoire National des Arts et Métiers, Chaire Santé et Développement, Paris, France; 9 Unité d'Epidémiologie des Maladies Emergentes, Institut Pasteur, Paris, France; 10 Service de Maladies infectieuses et tropicales, Hôpital Bichat Claude Bernard, Paris, France

**Keywords:** Delphi method, Expert knowledge elicitation, Methodological bias, Risk perception, Cognitive bias, HCV in Egypt

## Abstract

**Background:**

Data on HCV-related cirrhosis progression are scarce in developing countries in general, and in Egypt in particular. The objective of this study was to estimate the probability of death and transition between different health stages of HCV (compensated cirrhosis, decompensated cirrhosis and hepatocellular carcinoma) for an Egyptian population of patients with HCV-related cirrhosis.

**Methods:**

We used the “elicitation of expert opinions” method to obtain collective knowledge from a panel of 23 Egyptian experts (among whom 17 were hepatologists or gastroenterologists and 2 were infectiologists). The questionnaire was based on virtual medical cases and asked the experts to assess probability of death or probability of various cirrhosis complications. The design was a Delphi study: we attempted to obtain a consensus between experts via a series of questionnaires interspersed with group response feedback.

**Results:**

We found substantial disparity between experts’ answers, and no consensus was reached at the end of the process. Moreover, we obtained high death probability and high risk of hepatocellular carcinoma. The annual transition probability to death was estimated at between 10.1% and 61.5% and the annual probability of occurrence of hepatocellular carcinoma was estimated at between 16.8% and 58.9% (depending on age, gender, time spent in cirrhosis and cirrhosis severity).

**Conclusions:**

Our results show that eliciting expert opinions is not suited for determining the natural history of diseases due to practitioners’ difficulties in evaluating quantities. Cognitive bias occurring during this type of study might explain our results.

## Background

The Delphi method is used to reach a consensus between experts on a specific topic via a series of questionnaires interspersed with feedback of group answers [1]. Originally developed by the Rand Corporation for applications in forecasting the impact of technology on warfare [2], this method has since been applied to many fields, including medical research [3-6], in order to compensate for the lack of empirical data concerning specific topics in medical literature.

The aim of this method is not to collect knowledge about a subject, but rather to gather opinions [7]. This implies that erroneous estimates might occur in evaluation of some quantities by a panel of experts even when a convergence of opinion is observed. In particular, quantities such as probability of disease progression according to health status involve reasoning mechanisms that may produce cognitive bias. Here we demonstrate this, and we explain why the Delphi method may not be accurate when estimating probabilities of disease evolution. We use the example of a Delphi study we conducted in Egypt to estimate evolution of HCV-infected patients with cirrhosis.

The primary objective of this study was to use expert opinion to determine transition probabilities in a decision model of natural history of HCV-related cirrhosis in Egypt, namely, probability of death and probability of transition between the different stages of HCV (compensated cirrhosis, decompensated cirrhosis, hepatocellular carcinoma (HCC), etc.). Studies of the natural history of HCV-related cirrhosis have previously been published [8,9], but they focused on populations in northern countries with different genotypes (in Egypt, HCV infections are mainly genotype 4 [10], while in northern countries, this genotype is uncommon [11]); moreover, the Egyptian health care system differs, as does the population. In Egypt, the absence of alcohol is favorable to patients, while co-infection with bilharzias, hepatitis B or overweight, increase the risk of complications. For these reasons, we felt that literature estimations were inappropriate for an Egyptian population.

## Methods

### Delphi

The study was conducted according to the following plan:

1. Elaboration of the questionnaire: a questionnaire was designed to collect the estimated probability of evolution from cirrhosis to complications or death. This questionnaire was tested on two clinicians prior to the first-round meeting to ensure that questions were sufficiently clear for the reader.

2. Choice of experts: a panel of experts was selected according to two criteria. First, an equal proportion of professorial and non-professorial physicians, in order to have different profiles of experts and to gather information from different types and places of practice. Second, experts from different parts of Egypt, so as to avoid only specifically local information. We also chose experts with a wide range of ages and medical experience.

3. A private one-day face-to-face meeting was organized with experts so as to complete the questionnaire (first round): after receiving instructions via an explanation form (see Additional file 1) and an oral presentation of the study, each expert completed the questionnaire (see Additional file 2). No indications of possible likely values or clues were given and no preliminary discussion between experts took place at this stage. Experts also filled out a short form describing their medical specialty and experience and providing information about their familiarity with HCV infections. A numeric identifier was attributed to each participant to ensure anonymity of results.

4. A statistical summary of group answers was presented to the experts in the form of means with 95% confidence intervals and ranges for each quantity of interest. Experts were able to discuss the different questions and to eventually disagree concerning initial trends in responses. This was done at the end of the first-round meeting mentioned above.

5. Each participating expert’s questionnaire was returned to that expert with a summary of group responses. Experts were then asked to change their responses or to maintain their first choices based on what had been discussed (second round). Group answers in this final step constituted Delphi’s final results.

The study did not require formal ethical approval according to Egyptian regulations. The participants of this study provided informed consent for publication.

### Questionnaire

The questionnaire covered the natural history of HCV-related cirrhosis in Egyptian patients. Experts were asked to estimate the probability of death and/or HCC occurrence within one year starting from the following health stages: (i) compensated cirrhosis stage; (ii) first year of first cirrhosis decompensation episode (i.e. ascites, digestive hemorrhage, encephalopathy, icterus); (iii) stable decompensated cirrhosis stage (in the years following the first episode of decompensation without any other decompensation); and (iv) progressive decompensated cirrhosis stage (in the years following successive decompensation episodes). Moreover, they were asked to estimate the probability of death in patients with HCC. These transition probabilities from one health state to the next were estimated on the base of virtual cases according to the following patient characteristics: gender, age and time spent in current disease stage. The rank of each question is given in Table 1.

**Table 1:** Ranking of questions on the questionnaire

			**Gender = male**			**Gender = female**		
**From**	**Time already spent in this stage (years)**	**To**	**Age = 20**	**Age = 40**	**Age = 60**	**Age = 20**	**Age = 40**	**Age = 60**
Compensated cirrhosis		Death related to liver disease	1	2	3	36	37	38
Compensated cirrhosis	1-10	HCC	4	5	6	39	40	41
Compensated cirrhosis	>10	HCC	7	8	9	42	43	44
First decompensation	≤ 1	Death related to liver disease	10	11	12	45	46	47
First decompensation	≤ 1	HCC	13	14	15	48	49	50
Stable decompensated state		Death related to liver disease	16	17	18	51	52	53
Stable decompensated state	1-10	HCC	19	20	21	54	55	56
Stable decompensated state	>10	HCC		22	23		57	58
Progressive decompensated state		Death related to liver disease	24	25	26	59	60	61
Progressive decompensated state		HCC	27	28	29	62	63	64
HCC	≤1	Death related to liver disease	30	31	32	65	66	67
HCC	> 1	Death related to liver disease	33	34	35	68	69	70

Experts were able to respond to each question by specifying: (i) a single point-estimate; or (ii) a range with a lower and higher estimate. The questionnaire is available in the Additional file 2.

### Statistical analysis

A descriptive analysis was performed to summarize answers from each round. Means, 95% confidence intervals (95% CI) and interquartile ranges (IQR) for each value were estimated. When the expert’s answer was a range rather than a point estimate, we chose the value in the middle of this range as the point estimate of probability. Agreement between experts was measured by the intraclass correlation coefficient (ICC) with 95% confidence interval [12].

Analyses were performed with SAS software version 9.2 (SAS Institute, Cary, NC). ICC was calculated using the %INTRACC macro written by R.M. Hamer (http://www.psych.yorku.ca/lab/sas/intracc.htm).

## Results

### Characteristics of the panel of experts

A panel of 23 experts participated in the two Delphi rounds. Seventeen out of 23 participants were hepatologists, 2 were infectiologists and 4 did not report their medical specialty. Median age of participants was 38 years of age (IQR: 34–53), with an average of 15 years of medical practice (IQR: 10–24). All experts except two, for whom this variable was missing, reported that at least 10% of their patients were HCV-positive. Most experts were from Cairo (18/23 - 78%). Others were from Ismaïlia (2/23 - 9%), Banha (2/23 - 9%) and Tanta (1/23 - 4%).

### Estimates of transition probabilities

To estimate transition probabilities, for 33% of the questions, experts gave a range of values in response to questions. For other questions, they responded by choosing a single value.

**Figure 1:**
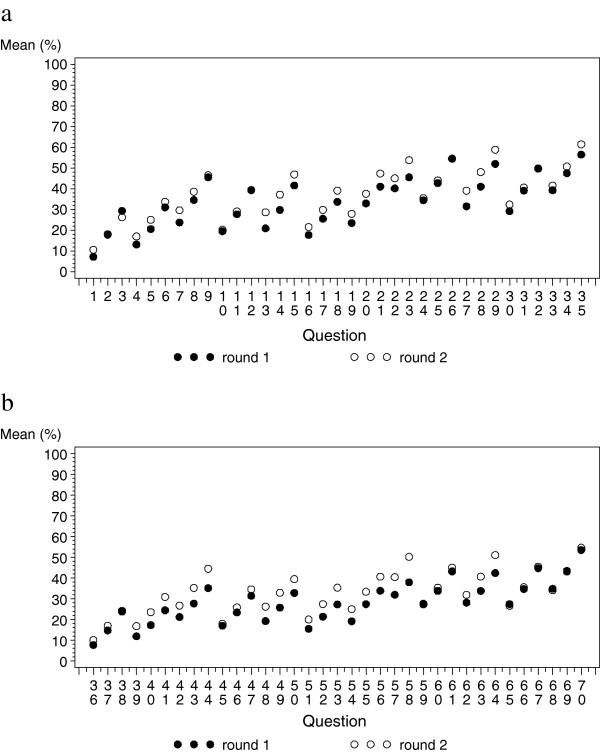
**Mean estimation of transition probabilities by round for men (a) and women (b).** Questions are ranked by current stage, transition stage and age. Corresponding questions are found in Table 1.

Table 2 presents values of mean transition probabilities estimated by the expert panel at the end of the second round with 95% confidence intervals. Figure 1 represents mean transition probabilities for each question at the first and second round in the same order as in the questionnaire. A logical trend was observed for each value: risks increased with age. Moreover, the mean probability of transition (corresponding to death probability or aggravation of liver disease probability) was consistently higher for men than for women, although the difference was not always significant. In addition, probability of death increased with the different stages of the disease: probability of death was between 10.1% and 26.4% in compensated cirrhosis, between 18.0% and 39.4% in case of first decompensation, between 20.0% and 39.2% for a stable decompensated state, between 27.6% and 54.5% for a progressive decompensated state and between 26.6% and 61.5% for patients with HCC. The same type of gradation was observed for risk of occurrence of HCC.

**Table 2:** **Main results of the second round of Delphi - mean transition probabilities (%) with 95**% **CI estimated at the second round of Delphi, and comparison with results from the literature (italics)**[8,9,13,14]

			**Gender = male**			**Gender = female**		
**Transition probability from**	**Time already spent in this stage (years)**	**To**	**Age = 20**	**Age = 40**	**Age = 60**	**Age = 20**	**Age = 40**	**Age = 60**
Compensated cirrhosis	< 1	Death related to liver disease	10.7% (8.3% - 13.0%)	18.2% (13.9% - 22.5%)	26.4% (19.9% - 32.8%)	10.1% (7.4% - 12.8%)	17% (12.9% - 21.1%)	24.3% (18.2% - 30.3%)
		[8]	*1*%	*1*%	*1*%	*1*%	*1*%	*1*%
Compensated cirrhosis	1 - 10	HCC	17.1% (13.3% - 20.9%)	25.1% (19.8% - 30.4%)	33.8% (26.2% - 41.4%)	16.8% (11.8% - 21.9%)	23.6% (18.0% - 29.1%)	30.9% (23.6% - 38.2%)
		[9]	*1*%	*1*%	*3*%	*0.4*%	*0.4*%	*1.2*%
Compensated cirrhosis	>10	HCC	29.7% (22.2% - 37.2%)	38.7% (30.0% - 47.4%)	46.6% (37.4% - 55.8%)	26.7% (19.1% - 34.3%)	35.2% (26.5% - 44.0%)	44.5% (34.7% - 54.3%)
		[9]	*2*%	*2*%	*4.5*%	*0.6*%	*0.6*%	*1.8*%
First decompensation	≤ 1	Death related to liver disease	20.3% (14.9% - 25.8%)	29.1% (22.8% - 35.5%)	39.4% (31.6% - 47.1%)	18.0% (13.3% - 22.7%)	25.9% (19.9% - 31.9%)	34.6% (27.5% - 41.7%)
		[8]	*39*%	*39*%	*39*%	*39*%	*39*%	*39*%
First decompensation	≤ 1	HCC	28.7% (20.3% - 37.1%)	37.2% (28.6% - 45.9%)	47.0% (37.4% - 56.5%)	26.2% (18.7% - 33.7%)	32.9% (24.7% - 41.1%)	39.5% (30.2% - 48.9%)
Stable decompensated state^*^		Death related to liver disease	21.6% (16.2% - 27.0%)	29.9% (23.8% - 35.9%)	39.2% (32.1% - 46.2%)	20% (14.6% - 25.4%)	27.5% (21.2% - 33.8%)	35.4% (28.1% - 42.7%)
		[8]	*12.5*%	*12.5*%	*12.5*%	*12.5*%	*12.5*%	*12.5*%
Stable decompensated state^*^	1 - 10	HCC	28.0% (21.5% - 34.4%)	37.7% (30.2% - 45.1%)	47.4% (39.1% - 55.7%)	25.1% (18.5% - 31.6%)	33.4% (26.5% - 40.4%)	40.7% (32.6% - 48.8%)
		[9]	*1*%	*1*%	*3*%	*0.4*%	*0.4*%	*1.2*%
Stable decompensated state^*^	>10	HCC		45.1% (35.1% - 55.1%)	53.9% (42.9% - 64.9%)		40.5% (31.5% - 49.4%)	50.3% (39.5% - 61.1%)
		[9]		*2*%	*4.5*%		*0.6*%	*1.8*%
Progressive decompensated state^*^		Death related to liver disease	35.6% (26.9% - 44.2%)	44.1% (35.5% - 52.8%)	54.5% (44.2% - 64.9%)	27.6% (20.4% - 34.9%)	35.3% (28.0% - 42.7%)	45.0% (36.3% - 53.8%)
		[8]	*15.6*%	*15.6*%	*15.6*%	*15.6*%	*15.6*%	*15.6*%
Progressive decompensated state^*^		HCC	39.1% (29.4% - 48.9%)	48.1% (37.9% - 58.3%)	58.9% (47.5% - 70.4%)	31.9% (23.8% - 40.0%)	40.7% (31.7% - 49.8%)	51.0% (40.8% - 61.3%)
HCC	≤ 1	Death related to liver disease	32.5% (25.6% - 39.3%)	40.7% (33.3% - 48.2%)	49.9% (41.5% - 58.4%)	26.6% (18.8% - 34.5%)	35.6% (27.5% - 43.8%)	45.5% (36.4% - 54.6%)
		[9]	*80*%	*80*%	*85*%	*80*%	*80*%	*85*%
HCC	> 1	Death related to liver disease	41.6% (33.9% - 49.3%)	50.8% (42.5% - 59.1%)	61.5% (52.1% - 70.9%)	34.1% (26.1% - 42.2%)	43.5% (35.0% - 51.9%)	54.6% (45.1% - 64.2%)
		[9]	*35*%	*35*%	*35*%	*35*%	*35*%	*35*%

^*^We assumed that Child-Pugh score class B corresponds to stable decompensated cirrhosis and that class C corresponds to a progressive decompensated state.

**Figure 2:**
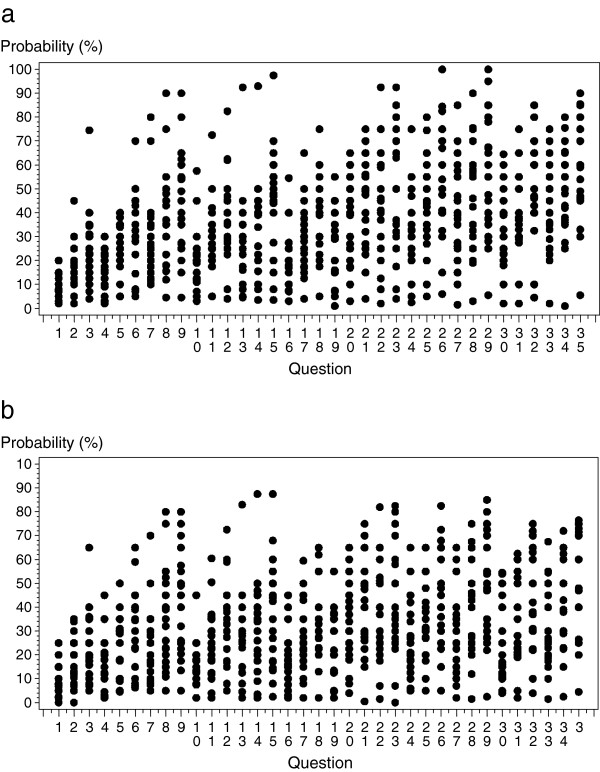
**Scatter plot of individual transition probability estimates after the second round of Delphi for men (a) and women (b).** Corresponding questions are found in Table 1.

However, confidence intervals surrounding transition probabilities were found to be wide. For example, widths of intervals ranged from 4.7% to 12.9% for death probability in compensated cirrhosis (first year), from 10.8% to 14.6% for death probability in stable decompensated cirrhosis and from 14.7% to 20.7% for death probability in a progressive decompensated state, all ages and genders confounded. Scatter plots in Figure 2 show responses of each participant as a function of each question. It illustrates the general wide variability of participant responses to the different questions; variations in responses for question 15, for example, ranged from 5% to 90%.

Based on the intraclass correlation coefficient, variability between experts for the entire questionnaire was estimated at 0.33 [95% CI: 0.26-0.43] for the first round and 0.51 [95% CI: 0.43-0.61] for the second. Although relative convergence appeared to occur between the two rounds, disagreement remained high. The minimum expected value for the intraclass correlation coefficient with high agreement between participants was evaluated at 0.75 [12].

**Figure 3:**
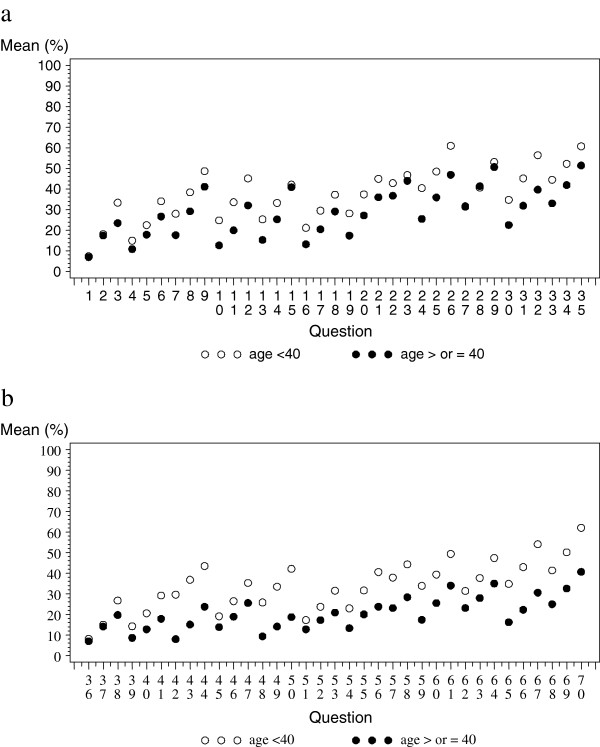
**Mean probability of transition at the end of the second round for men (a) and women (b) by age group.** White circles represent ≥40 age group and black dots represent the <40 age group.

We performed an analysis stratifying experts by age: <40 years of age (13/23) vs. ≥ 40 (10/23). Figure 3 presents results of this analysis with mean probabilities of transition for men (a) and women (b) estimated at the end of the second round. White circles represent the mean answer for the <40-year-old group and black dots for the ≥ 40-year-old group. A difference seemed to emerge between the two subgroups of experts. Younger experts were, in general, more pessimistic, with higher probabilities of progression of liver disease and death. In addition, estimates of the intraclass coefficient differed between the two groups, although they were not statistically significant (ICC for the < 40-year-old group: 0.54 [CI 95%: 0.47-0.65]; ICC for the ≥ 40-year-old group: 0.46 [CI 95%: 0.37-0.56]).

## Discussion

There exist no data on the natural history of HCV disease in developing countries, and Egypt is no exception. We therefore conducted this Delphi analysis to specifically estimate progression of patients with cirrhosis to HCC and death in that country.

The natural history of HCV disease has been estimated for developed countries. We assume that, in Egypt, management of HCV-infected patients with cirrhosis differs from that of developed countries. Moreover, patient characteristics and co-morbidities are not necessarily similar in these settings, and it was for these reasons that we conducted our study. However, we were surprised at differences in estimations in our Delphi study compared to those reported in developing countries [8,9,13,14]. Transition probabilities for some stages and, in particular, early stages of cirrhosis, seemed unexpectedly high in our study (i.e. annual death probabilities for patients with compensated cirrhosis were 10- to 24-fold higher in our study than that found in the medical literature in developed countries [8]). In contrast, transition probabilities for other stages such as late stages of cirrhosis seemed to be lower in our study (i.e. annual death probability for patients with first cirrhosis decompensation were 1- to 2-fold lower in our study [8]).

Moreover, we found very strong variability between the responses of the different participants. Certain transition probability estimates ranged from 5% to 90%. In addition, the intraclass correlation coefficient estimating overall degree of agreement between experts was low. This lack of consensus calls into question the estimates obtained by the Delphi process, especially as other studies were conducted in the past to estimate transition probabilities by eliciting expert opinions, with similar results. Soares *et al.* attempted to estimate transition probabilities for a cost effectiveness transition model of negative pressure wound therapy for severe pressure ulceration [15]. The authors observed variability in experts’ answers, and felt that this type of result is desirable, since it ensures that all views are represented. However, the design of that exercise was not a Delphi study, which specifically aims to obtain a consensus between experts. Schultz *et al.* applied the Delphi technique to lung cancer progression [6], with estimation of 5-year survival probabilities by 14 experts. They did not make any comparison with expected values for this disease because of the absence of empirical data, but high variability in responses between experts was observed. The authors concluded that the different beliefs may explain variations between practitioners in management of patients with solitary pulmonary nodules. They mentioned small sample size as one of the study limitations and stated that such a result – that is, variability in opinions – may not be generalized. Lubell *et al.* conducted a Delphi survey to estimate transition probabilities in the natural history and medical management of malaria and acute febrile illness [5]. Twenty-one panellists participated in that study. They too noted wide dispersion on several questions, ranging from 5% to 100%, and overall lack of agreement between experts. Our survey suggests that this type of variability can be found for other diseases. This is worrisome, since erroneous perception of risk of disease progression by practitioners may influence medical choices concerning these diseases as well. In a broader context, Cahan *et al.* were interested in the estimation of probabilities by physicians in the context of a “threshold approach” for decision-making in medicine [16,17]. They asked physicians to assess the probability of various diagnoses from a case description in a single anonymous questionnaire. Their results indicated that practitioners generally overestimate the probabilities of each diagnosis, resulting in a total of more than 100% (“subadditivity”) for a non-exhaustive list of mutually exclusive diagnoses [17]. Second, they also found a wide variability in responses among experts [16,17]. They did not use a Delphi process, which specifically aims to increase agreement between the responders. However, the same type of problem is observed. The authors highlighted the “support theory” of Tversky and Koehler [18,19]. This theory claims that the probability assigned to a description of an event depends on the description: two different descriptions of the same event can lead to different estimates. Results of these studies show that it is indeed a lack of probabilistic thinking rather than the type of probability, or a lack of observation of specific cases, that may be involved.

Other explanations may be proposed for the wide variability in responses and errors in transition probability estimations. First, the infrequency of events and the rarity of profiles may lead to imaginary rather than statistical estimates (i.e. cognitive-based estimates) [20]. For example, for patients with compensated cirrhosis, orders of magnitude for probabilities are around 1 percent and sometimes 1 per mile, and overestimations are clearly accentuated for such a state. Under such conditions, we presume that events are not frequent enough to be familiar to practitioners. Profiles may be too specific to allow practitioners a good representation of the situation described in the questionnaire. Other potential explanations concerning cognitive bias and perception of risk are present in the literature [21,22]. Kahneman *et al.* and Tversky *et al.*, in particular, describe several cognitive biases and thought mechanisms involved in human judgment. The “representativeness heuristic” implies that probability estimations by humans are not based on statistical or probabilistic reasoning, but rather on judgment by representativeness, i.e. on stereotypes based on the description of patient characteristics. This mode of reasoning leads to erroneous evaluations, which are sometimes in contradiction with elementary probabilistic properties [23]. Other cognitive biases may occur because of the “availability heuristic”, which states that reasoning tends to be based on immediately available information. Information availability may be influenced by various factors. Tversky *et al.* suggest – among others – salience: “it is a common experience that the subjective probability of traffic accidents rises temporarily when one sees a car overturned by the side of the road” [22]. It is reasonable to assume that a similar cognitive bias occurs when we ask physicians to estimate event probabilities regarding their patients’ deaths. Abstraction also has an impact on availability, because abstract quantities – such as probabilities – are not immediately available and require reasoning. Evaluation of such quantities can cause what Tversky *et al.* call “biases of imaginability” [22]: instances are generated according to a given rule. In our study, questions were ordered by gender, current stage of cirrhosis, transition stage and age (Table 1). Linearity of the different probability functions of age in Figure 1 suggests that experts responded according to a predetermined model (linear increase of risk with age) and not according to their practical experience.

In the Delphi survey, the iteration process can theoretically be repeated in order to increase the opinion’s convergence between experts. However, this point of view does not create a consensus [24], because of the risk of an artificial consensus: Delphi increases judgment convergence, but not accuracy [1,20]. In our study, the relative convergence observed between the two rounds does not seem to lead responses toward more realistic values. In contrast, overestimated values become higher (Figure 1). If experts have no idea of the answer at the first round, there is no reason why iteration would lead to better estimations. In addition, anonymity might encourage experts to answer even if they are uncertain [25]. In our study, the mean number of missing values decreased between the two rounds, from 2.1 (9%) to 0.4 (2%). It is reasonable to assume that the difference might be explained by some form of group pressure.

Results mentioned above suggest that recommendations be made for solicitation of expert opinions. Delphi might be a useful tool for compensating for the lack of empirical data in medical research [26]. However, for Dalkey, knowledge is more reliable than opinion [7] and this is why, in the presence of available data, Delphi is a useless technique: at best, results are consistent with empirical results; if results differ, then empirical estimations are a priori better. Moreover, the nature of the information requested must be taken into account. While it is expected that information concerning medical practices is easily evaluated by a panel of experts, probabilistic measurements or abstract information can cause cognitive bias in estimations [21,22]. Thus, it is recommended that Delphi not be used to obtain information on the natural history of a disease or survival probabilities. Finally, the relevance of the questions is subject to caution. Questions that are too precise could paradoxically be counterproductive, and the familiarity of experts with the different aspects of the questionnaire should be subjected to preliminary discussion.

Our study suffers from several limitations. First, data on transition probabilities used to evaluate quality of results were taken from studies in northern countries. Second, alternative formulations of questions may have been more appropriate for estimating transition probabilities by experts without probabilistic background [15,27]. Finally, opinion (unlike knowledge) is imperfect and this uncertainty must be measured in the questionnaire [28]. We left two options to the experts when responding to questions: either respond by a single value or, if unable to provide a single value, respond by giving a range that takes uncertainty into account. The fact that only 33% of the questions were answered in the form of an interval illustrates the fact that we may have failed to capture this individual uncertainty [15,27].

## Conclusion

Elicitation of expert opinions to determine transition probabilities between different health stages of HCV natural history in Egypt seems to be inexact, with substantial disparity between experts. Estimation of disease progression probabilities by practitioners involves cognitive biases that imply a lack of reliability of the estimates. Thus, even though elicitation of expert opinion is a relatively easy way of compensating for a lack of empirical data, it does not seem suitable for estimation of probabilities of medical events.

## Abbreviations

CI: Confidence interval; HCC: Hepatocellular carcinoma; HCV: Hepatitis C virus; ICC: Intraclass correlation coefficient; IQR: Interquartile range.

## Competing interests

SDB received grants from Roche, Janssen-Cilag and Schering-Plough and consultancy honoraria from Merck and GlaxoSmithKline. GE received funds from BioGenesic Phasma and BMS. YY received travel grants, honoraria for presentations at workshops and consultancy honoraria from Abbott, Bristol-Myers Squibb, Gilead, Merck, Roche, Tibotec and ViiV Healthcare. None of the other authors report any association that might pose a conflict of interest.

## Authors’ contributions

YY had the idea for the study. DO, SBD, VC and YY contributed to the conception and design of the analysis. GE and MKM took part in the selection of experts. GE, MEK, MES, and MKM were part of the panel of experts. AM, DO, and AC performed first-round statistical analysis during the Delphi process. AC performed the second round and additional statistical analysis. All authors contributed to interpretation of data. AC drafted the article and all authors critically revised it for important intellectual content. All authors approved the final version of the manuscript to be published.

## Pre-publication history

The pre-publication history for this paper can be accessed here:

http://www.biomedcentral.com/1471-2288/14/39/prepub

## Supplementary Material

Additional file 1 Explanation Form. Click here for file

Additional file 2 Questionnaire. Click here for file
