# Supplementary material for: Is expert opinion reliable when estimating transition probabilities? The case of HCV-related cirrhosis in Egypt
Source: BMC Med Res Methodol. 2014 Mar 17;14:39. doi: 10.1186/1471-2288-14-39 (PMC4003824; doi:10.1186/1471-2288-14-39)
Supplement: Additional file 1 — Explanation Form. [file 1471-2288-14-39-S1.pdf]

EXAMPLE AND DEFINITIONS

Example

In this example, we are asked to anticipate the risk of death from HCC during the coming year for a man, 20 years old, who was diagnosed with a compensated cirrhosis 1 year before… >>>>

Sex	Age of the patient (years)	Disease stage of the patient	Time already spent in this stage (years)	Put a mark in the next column to show the probability that next year the patient will be ...
Man	20	Compensated cirrhosis	1	dead, related to liver disease

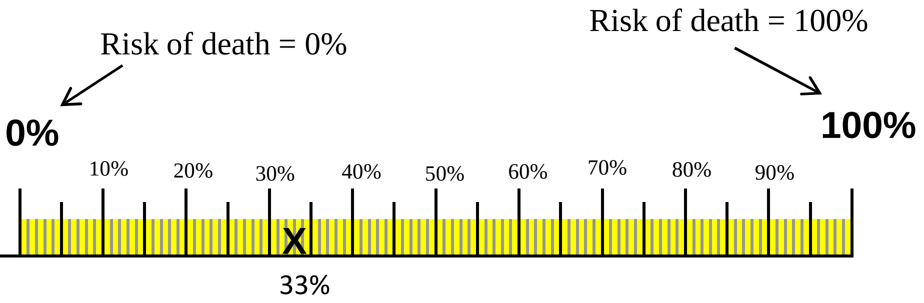

If you don't know exactly the risk, you can give a range
Example:

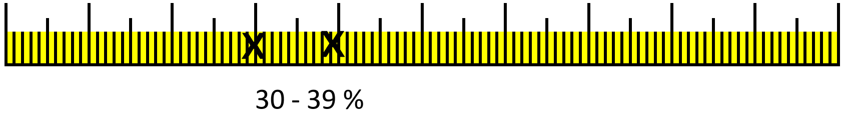

Definitions :

First decompensation: means the first decompensation episode (ascites, digestive hemorrhage, encephalopathy, icterus)

Stable decompensated state: means the patient has a history of a first decompensation, but after the first one he doesn't do any other decompensation episode

Progressive decompensated state: means the patient has successive decompensation episodes

HCC: hepatocellular carcinoma
