# Supplementary material for: Is expert opinion reliable when estimating transition probabilities? The case of HCV-related cirrhosis in Egypt
Source: BMC Med Res Methodol. 2014 Mar 17;14:39. doi: 10.1186/1471-2288-14-39 (PMC4003824; doi:10.1186/1471-2288-14-39)
Supplement: Additional file 2 — Questionnaire. [file 1471-2288-14-39-S2.pdf]

APPENDIX: QUESTIONNAIRE

COMPENSATED CIRRHOSIS

Gender	Age of the patient (years)	Disease stage of the patient	Time already spent in this stage (years)	Put a mark in the next column to show the probability that next year the patient will be/have ...	0% — 100%
Man	20	Compensated cirrhosis		dead, related to liver disease	
Man	40	Compensated cirrhosis		dead, related to liver disease	
Man	60	Compensated cirrhosis		dead, related to liver disease	

Gender	Age of the patient (years)	Disease stage of the patient	Time already spent in this stage (years)	Put a mark in the next column to show the probability that next year the patient will be/have ...	0% — 100%
Man	20	Compensated cirrhosis	1-10	HCC	
Man	40	Compensated cirrhosis	1-10	HCC	
Man	60	Compensated cirrhosis	1-10	HCC	
Man	20	Compensated cirrhosis	>10	HCC	
Man	40	Compensated cirrhosis	>10	HCC	
Man	60	Compensated cirrhosis	>10	HCC	

FIRST DECOMPENSATION*

Gender	Age of the patient (years)	Disease stage of the patient	Time already spent in this stage (years)	Put a mark in the next column to show the probability that next year the patient will be/have ...	0% — 100%
Man	20	First decompensation	≤ 1	dead, related to liver disease	
Man	40	First decompensation	≤ 1	dead, related to liver disease	
Man	60	First decompensation	≤ 1	dead, related to liver disease	

					0% — 100%
Man	20	First decompensation	≤ 1	HCC	
Man	40	First decompensation	≤ 1	HCC	
Man	60	First decompensation	≤ 1	HCC	

* First decompensation: means the first decompensation episode (ascites, digestive hemorrhage, encephalopathy, icterus)

STABLE DECOMPENSATED STATE*

Gender	Age of the patient (years)	Disease stage of the patient	Time already spent in this stage (years)	Put a mark in the next column to show the probability that next year the patient will be/have ...	0% — 100%
Man	20	Stable decompensated state		dead, related to liver disease	
Man	40	Stable decompensated state		dead, related to liver disease	
Man	60	Stable decompensated state		dead, related to liver disease	
					0% — 100%
Man	20	Stable decompensated state	1-10	HCC	
Man	40	Stable decompensated state	1-10	HCC	
Man	60	Stable decompensated state	1-10	HCC	
Man	40	Stable decompensated state	>10	HCC	
Man	60	Stable decompensated state	>10	HCC	

* Stable decompensated state: means the patient has a history of first decompensation, but afterwards no further decompensation episode

PROGRESSIVE DECOMPENSATED STATE*

Gender	Age of the patient (years)	Disease stage of the patient	Time already spent in this stage (years)	Put a mark in the next column to show the probability that next year the patient will be/have ...	0% — 100%
Man	20	Progressive decompensated state		dead, related to liver disease	
Man	40	Progressive decompensated state		dead, related to liver disease	
Man	60	Progressive decompensated state		dead, related to liver disease	
					0% — 100%
Man	20	Progressive decompensated state		HCC	
Man	40	Progressive decompensated state		HCC	
Man	60	Progressive decompensated state		HCC	

* Progressive decompensated state: means the patient has successive decompensation episodes

HCC*

Gender	Age of the patient (years)	Disease stage of the patient	Time already spent in this stage (years)	Put a mark in the next column to show the probability that next year the patient will be...	0% — 100%
Man	20	HCC	≤ 1	dead, related to liver disease	
Man	40	HCC	≤ 1	dead, related to liver disease	
Man	60	HCC	≤ 1	dead, related to liver disease	

Gender	Age of the patient (years)	Disease stage of the patient	Time already spent in this stage (years)		0% — 100%
Man	20	HCC	> 1	dead, related to liver disease	
Man	40	HCC	> 1	dead, related to liver disease	
Man	60	HCC	> 1	dead, related to liver disease	

* HCC: hepatocellular carcinoma

COMPENSATED CIRRHOSIS

Gender	Age of the patient (years)	Disease stage of the patient	Time already spent in this stage (years)	Put a mark in the next column to show the probability that next year the patient will be/have ...	0% – 100%
Woman	20	Compensated cirrhosis		dead, related to liver disease	
Woman	40	Compensated cirrhosis		dead, related to liver disease	
Woman	60	Compensated cirrhosis		dead, related to liver disease	
Woman	20	Compensated cirrhosis	1-10	HCC	
Woman	40	Compensated cirrhosis	1-10	HCC	
Woman	60	Compensated cirrhosis	1-10	HCC	
Woman	20	Compensated cirrhosis	>10	HCC	
Woman	40	Compensated cirrhosis	>10	HCC	
Woman	60	Compensated cirrhosis	>10	HCC	

FIRST DECOMPENSATION*

Gender	Age of the patient (years)	Disease stage of the patient	Time already spent in this stage (years)	Put a mark in the next column to show the probability that next year the patient will be/have ...	0% ... 100%
Woman	20	First decompensation	≤ 1	dead, related to liver disease	
Woman	40	First decompensation	≤ 1	dead, related to liver disease	
Woman	60	First decompensation	≤ 1	dead, related to liver disease	

					0% ... 100%
Woman	20	First decompensation	≤ 1	HCC	
Woman	40	First decompensation	≤ 1	HCC	
Woman	60	First decompensation	≤ 1	HCC	

* First decompensation: means the first decompensation episode (ascites, digestive hemorrhage, encephalopathy, icterus)

STABLE DECOMPENSATED STATE*

Gender	Age of the patient (years)	Disease stage of the patient	Time already spent in this stage (years)	Put a mark in the next column to show the probability that next year the patient will be/have ...	0% — 100%
Woman	20	Stable decompensated state		dead, related to liver disease	
Woman	40	Stable decompensated state		dead, related to liver disease	
Woman	60	Stable decompensated state		dead, related to liver disease	
Woman	20	Stable decompensated state	1-10	HCC	
Woman	40	Stable decompensated state	1-10	HCC	
Woman	60	Stable decompensated state	1-10	HCC	
Woman	40	Stable decompensated state	>10	HCC	
Woman	60	Stable decompensated state	>10	HCC	

* Stable decompensated state: means the patient has a history of first decompensation, but afterwards no further decompensation episode

PROGRESSIVE DECOMPENSATED STATE*

Gender	Age of the patient (years)	Disease stage of the patient	Time already spent in this stage (years)	Put a mark in the next column to show the probability that next year the patient will be/have ...	0% — 100%
Woman	20	Progressive decompensated state		dead, related to liver disease	
Woman	40	Progressive decompensated state		dead, related to liver disease	
Woman	60	Progressive decompensated state		dead, related to liver disease	
					0% — 100%
Woman	20	Progressive decompensated state		HCC	
Woman	40	Progressive decompensated state		HCC	
Woman	60	Progressive decompensated state		HCC	

* Progressive decompensated state: means the patient has successive decompensation episodes

HCC*

Gender	Age of the patient (years)	Disease stage of the patient	Time already spent in this stage (years)	Put a mark in the next column to show the probability that next year the patient will be...	0% ... 100%
Woman	20	HCC	≤ 1	dead, related to liver disease	
Woman	40	HCC	≤ 1	dead, related to liver disease	
Woman	60	HCC	≤ 1	dead, related to liver disease	

					0% ... 100%
Woman	20	HCC	> 1	dead, related to liver disease	
Woman	40	HCC	> 1	dead, related to liver disease	
Woman	60	HCC	> 1	dead, related to liver disease	

* HCC: hepatocellular carcinoma
